# Tincture of Opium and Laudanum

**Published:** 1915-09-11

**Authors:** 


### The Hospital

The Workers' Newspaper of Administrative Medicine and Institutional Life, Administration, National Insurance and Health.

No. 1526, Vol. LVIII. SATURDAY, SEPTEMBER 11, 1915. PRICE ONE PENNY

### TINCTURE OF OPIUM AND LAUDANUM.

A communication from the Privy Council, and considered at a recent meeting of the Executive Committee of the General Medical Council, announces the emergence of a highly important and practical point relative to the sale to the public of certain preparations of opium. In essence the question to be considered is whether the sale of tincture of opium of the strength of the British Pharmacopœia of 1898 can be continued, in view of the altered strength of this preparation as effected by the Pharmacopœia of 1914. The latter preparation is stronger by one-third than the 1898 tincture, and hence, if the safety of the public is to be conserved, it is obvious there must be no risk of confusion between the two.

The circumstances which have brought the question to an issue are briefly as follows: Dr. F. J. Waldo, His Majesty's Coroner for the City of London, in his last annual report to the Lord President of the Privy Council, stated that chemists are selling under the name tincture of opium or laudanum preparations which differ in strength the one from the other. A copy of Dr. Waldo's report was sent by the Lord President to the Council of the Pharmaceutical Society, and the Council in replying enclosed a resolution adopted by them in January last. In this resolution it is recognised that when "laudanum," without any qualification, is asked for, the 1914 tincture must be supplied, but when "the 1898 preparation is demanded great care should be taken to label it accordingly, and the attention of the purchaser should be called to the fact that it is the 1898 preparation." It is the position so presented which calls for consideration, and it must be admitted that certain difficulties attend it.

There are some admissions which are common ground to all the parties concerned. Thus it is agreed that "tincture of opium" now means, and means only, the stronger tincture of the Pharmacopœia of 1914. The same is true of the term "laudanum," since this name appears as an official synonym in the Pharmacopœia. Either of these terms, whether written in a prescription or presented by a customer, requires therefore an interpretation which is quite beyond dispute. This interpretation is the tincture of the 1914 Pharmacopœia. What the Pharmaceutical Society proposes is that in certain circumstances "laudanum" shall possess another meaning—namely, that attached to it in the Pharmacopœia of 1898, and operative until the new issue of 1914. The question may possibly have a legal bearing, but our concern with it is restricted to its relation to the safety and welfare of the public. On this ground we conclude that the contention of the Pharmaceutical Society ought not to be sanctioned.

From what has already been said it is evident that the discussion does not include any difference of opinion in reference to the interpretation of physicians' prescriptions. Physicians, equally with pharmacists, are bound to use pharmacopœial terms in their pharmacopœial meaning, and when they write any of these terms, tincture of opium for example, they imply the standard now legally in force. But it is difficult to raise the public to this new standard. Wisely or unwisely, the public has been accustomed to purchase "laudanum" and to use it as a local sedative for the relief of toothache and other pains; and there are persons who have accustomed themselves to take the drug in fairly considerable quantities. It is here that the pharmacist is placed in a position of difficulty. To say, as some, that he ought not to sell opium or its preparations to the public is to call upon him to accept a responsibility which the law does not impose. All that the law says is that these substances must only be sold if certain conditions are satisfied. Granted this, the pharmacist has no legal duty either to interrogate or to instruct the purchaser. But if he now supplies to the public a preparation one-third stronger than the public has been accustomed to purchase and to use, it is manifest that the transaction carries some measure of risk. This risk may be met, or an attempt to meet it may be made, by explanation of the altered conditions. But every customer is not tolerant of explanations, and not a few will continue to demand the preparation which they have been accustomed to obtain. In addition, the new tincture is of such a strength that anyone purchasing it must sign the "Poisons Book," while the weaker preparation of 1898 escaped this formality. Here, again, as is easily seen, there may come objections from the customer and an insistence upon a desire for the "laudanum" which avoids the necessity of "signing the book." It is impossible in these circumstances to avoid some measure of sympathy with the worried pharmacist. He is reproached by an obstinate and unreasonable customer, and is at the same time conscious that what he refuses a rival practitioner may be ready to grant. It is out of such circumstances as these, we imagine, that the resolution of the Pharmaceutical Society has taken shape.

Our objection to the Society's resolution, as we have already stated, is that its adoption would be inconsistent with the public safety, though, at the same time, we should question whether it is in harmony with the spirit of the national Pharmacopœia. Obviously there must be danger when "laudanum" may mean sometimes one preparation and sometimes another and different preparation. A customer, though once educated to the necessity of saying "laudanum 1898," may readily forget the qualification on a subsequent occasion, and with results disastrous to himself. Even if he himself appreciates the situation, will his messenger be equally alert? Again, it can hardly be proposed that permission to sell the 1898 tincture can be continued indefinitely, and hence the change must be made some time. Is it not wiser and safer to make it at once, even though the process may cause some inconvenience to the pharmacist and may excite some indignation in the customer? On the latter point there would be no concern were the rule of universal obligation, and no one will deny that it is well for the public to be compelled to satisfy conditions which tend to make the purchase of powerful drugs a serious undertaking. In any event, it is necessary to have a decision one way or the other, and in our opinion the safer course is to attach to "laudanum" the one meaning which the Pharmacopœia defines and to abolish the sale of the weaker tincture sanctioned by an authority which has now disappeared.

